# ﻿A new southern Atlantic cryptic marine shrimp species of *Acetes* (Decapoda, Sergestidae)

**DOI:** 10.3897/zookeys.1211.128059

**Published:** 2024-09-04

**Authors:** Gabriel L. Bochini, Rogério C. Costa, Fernando L. Mantelatto

**Affiliations:** 1 Laboratory of Bioecology and Crustacean Systematics (LBSC), Department of Biology, Faculty of Philosophy, Science and Letters at Ribeirão Preto (FFCLRP), University of São Paulo (USP), Av. Bandeirantes 3900, 14040-901, Ribeirão Preto (SP), Brazil University of São Paulo Ribeirão Preto Brazil; 2 Laboratory of Biology of Marine and Freshwater Shrimp (LABCAM), Department of Biological Sciences, School of Sciences, São Paulo State University (UNESP), Av. Eng. Luiz Edmundo Carrijo Coube, 14-01, 17033-360, Bauru (SP), Brazil São Paulo State University Bauru Brazil

**Keywords:** *Acetesamericanus*, Brazil, Cananéia, Dendrobranchiata, hidden diversity, new species, taxonomy

## Abstract

A recently published molecular phylogenetic analysis, focusing on selected Western Atlantic subspecies of *Acetesamericanus* Ortmann, 1893 and allies, was inconclusive about relationships among these members. This previous study found three groups that split into two distinct lineages: *Acetesamericanus* (Brazil 1) (= *A.americanus* sensu stricto) and *Acetesamericanus* (Brazil 2) + *A.americanus* (USA). Combined morphometry and molecular analyses applied to members of the group *Acetesamericanus* (Brazil 2) revealed a new unidentified species genetically related to the *A.americanus* representatives. However, at that time, no conclusive morphological characters were found to identify it. In the present study, following an in-depth morphological analysis of specimens from the three groups, including data on the type series and consideration of the subtle distinctions of members of each lineage, morphological features of the reproductive structures (petasma and genital sternite) were found to characterize the new species, which is formally described and named herein.

## ﻿Introduction

The genus *Acetes* H. Milne Edwards, 1830 is represented by 13 species worldwide (De Grave and Fransen 2011; DecaNet 2024). Only three species are distributed in the Western Atlantic: *Acetesamericanus* Ortmann, 1893, *Acetesmarinus* Omori, 1975 and *Acetesparaguayensis* Hansen, 1919 (Costa and Simões 2016; Simões et al. 2023). Historically, *Acetesamericanus* has presented taxonomic instability in four subspecies (*A.americanusamericanus* Ortmann, 1893; *A.a.carolinae* Hansen, 1933; *A.a.louisianensis* Burkenroad, 1934a; *A.a.limonensis* Burkenroad, 1934a), but only two of them are considered valid (Holthuis 1948) and accepted (see Simões et al. 2023 and DecaNet 2024 for review and details below), nowadays. *Acetesamericanus* features a wide geographic distribution in the Western Atlantic and presents two subspecies acknowledged for their geographic separation: *Acetesamericanuscarolinae* is distributed in North America and *Acetesamericanusamericanus*, in South America (DecaNet 2024). However, there are regions (Central America and Northern South America) where both subspecies are distributed in sympatry (Perez-Farfante and Kensley 1997). Despite the morphological similarity between these two subspecies, there are subtle morphological differences in their body and cornea lengths (Omori 1975). Yet, taxonomic inconsistencies were reported in both subspecies in the 1970s. Therefore, their geographically coexistent and subtle features make their validity doubtful and still unsolved. Accordingly, and due to pending future taxonomic rearrangements, the following nomenclature was adopted below: *A.americanus* sensu stricto - since the taxonomic status based on phylogenetic relationship, geographical distribution and morphology is clear [see lineage *A.americanus* (Brazil 1) in Simões et al. (2023); present study]; and *Acetesa.carolinae* - due to taxonomic uncertainties under this entity.

Specimens collected from the Brazilian coast were previously identified as *A.americanus* during a long-term biodiversity project focused on the Brazilian fauna, based on integrative analyses (see Mantelatto et al. 2018, 2022). These specimens showed some variability in morphological characters that have called our attention and presented some doubtful identifications. Recently, our team conducted a molecular study (Simões et al. 2023) to compare *A.americanus* specimens collected in South America to *A.a.carolinae* specimens sampled in North America. It was done using two mitochondrial markers to test the genetic validity of both subspecies and the likely existence of other entities distributed along the Western Atlantic that were not mentioned in previous investigations. This study found three strongly-supported groups divided into two different genetic lineages composed of *A.americanus* sensu stricto (Brazil 1) and *Acetesamericanus* (Brazil 2) + *A.americanus* (USA) (see Simões et al. 2023; figs 3–6). The aforementioned authors used additional morphometric analysis (see Simões et al. 2023; fig. 7) to corroborate the lineages and the new unrecognized species, ‘*Acetesamericanus* (Brazil 2)’, which was genetically related to *A.americanus* representatives.

In the present study, we formally describe *Acetesamericanus* (Brazil 2) based on morphology. Besides the significant support from previously developed DNA-based phylogenetic analyses, the new species was also compared to *Acetesa.americanus* and *A.a.carolinae*.

## ﻿Materials and methods

Specimens were collected under field permit approval by *Instituto Chico Mendes de Biodiversidade*/ICMBio, Protocol No. 23008-1, Permanent Licenses to RCC number 23012-4 and FLM 11777-2, and SISGEN CEA7CD5 and A5845DA. Most of them were deposited at the Crustacean Collection of the Department of Biology (**CCDB**), Faculty of Philosophy, Science and Letters at Ribeirão Preto, University of São Paulo (**FFCLRP/USP**). Additional loaned specimens and the designated type series are deposited in the following scientific collections: Zoology Museum of University of São Paulo, São Paulo, Brazil (**MZUSP**); Crustacean Collection of the Laboratory of Biology of Marine and Freshwater Shrimp, São Paulo State University (UNESP), Bauru, Brazil (**CCLC**); Crustacean Collection of Federal University of Rio Grande do Sul, Brazil (**DZ/UFRGS**); Crustacean Collection of Museu Nacional do Rio de Janeiro, Brazil (**MNRJ**); Oceanographic Museum of Federal University of Pernambuco, Brazil (**MOUFPE**); National Museum of Natural History, Smithsonian Institution, USA (**USNM**); University of Louisiana Zoological Collection, Lafayette, USA (**ULLZ**); and Natural History Museum of Denmark - University of Copenhagen, Denmark (**NHMD**).

The morphological description was based on characters and character states proposed by Omori (1975), D’Incao and Martins (2000) and Vereshchaka et al. (2016a, 2016b), which used the form of the genital area (thelycum) in females and the petasma shape in males as diagnostic characters. The phylogenetic positioning and topologies proposed by Simões et al. (2023) were followed to assess individuals’ morphology and identification.

Carapace length was measured from the rostrum tip to the carapace’s posterior margin and expressed in millimeters (mm). All measurements were taken with a calibrated ocular micrometer (+/− 0.1 mm) or digital caliper. Sex was assessed based on petasma (first pleopod) presence in males and on thelycum presence in females (Xiao and Greenwood 1993). Morphometric measurements and illustrations were carried out with the aid of a stereo microscope (Leica^®^ M205 C) coupled with a camera (Leica^®^ DFC 295), added with software Leica Application Suite version 3.8.0 for taking measurements. The resulting drawings were processed in Adobe Illustrator 2020^®^.

### ﻿Molecular analyzes

The phylogenetic hypothesis was created using the same sequences produced and deposited in GenBank by Simões et al. (2023) (Suppl. material 1). Maximum likelihood phylogenetic analyzes were performed using the IQ-TREE program (Miller et al. 2010) with the mitochondrial 16S Ribosomal RNA (16S rRNA) and cytochrome *c* oxidase subunit I (COI) genes concatenated. Branch support was assessed by ultrafast bootstrap with 1000 replications. *Acetesparaguayensis* Hansen, 1919 was included as an outgroup following the most recent global phylogeny (Vereshchaka 2017) and Simões et al. (2023). Intra- and interspecific genetic distances were estimated using MEGA 5.0 software (Tamura et al. 2011).

### ﻿Abbreviations

**cl** carapace length,

**coll(s).** collector(s),

**ind.** individuals,

**PL.** pleopods,

**coord.** coordinate.

## ﻿Results

### ﻿Taxonomy

**Superfamily Sergestoidea Dana, 1852**

**Family Sergestidae Dana, 1852**

**Genus *Acetes* H. Milne Edwards, 1830**

#### ﻿ Acetes maratayama

Taxon classificationAnimaliaDecapodaSergestidae

Bochini, Costa & Mantelatto sp. nov.

A8589B4B-FCE7-5824-B7F2-342352765F08

https://zoobank.org/BC6949CD-ABFE-48CA-8311-ED86CC7E5B6D

Figs 1 , 2 , 3 , 4

##### Type material.

***Holotype***: Brazil: • ♂ (cl 2.94 mm); CCDB 7957; São Paulo, Cananéia, Mar Pequeno; (24°59'55"S, 47°53'49"W); 5–10 m deep; colls. Costa, R.C. et al.; 17 April 2011. ***Paratypes***: • 4 ♂s and 4 ♀s (cl 2.70 – 3.93 mm); CCDB 7958 (photo available, one dissected specimen); same data as holotype • 1 ♂ and 1 ♀ (cl 2.9 and 4.04 mm, respectively); MOUFPE 22042; same data as holotype • 1 ♂ and 1 ♀ (cl 3.04 and 3.93 mm, respectively); MZUSP 45904; same data as holotype • 2 ♂s and 2 ♀s (cl 4.01 – 5.19 mm); CCDB 7959; Brazil, Rio de Janeiro, Macaé; (22°22'13.65"S, 41°39'9.42"W); colls. Davanso, T.M. et al.; 01 September 2013 • 2 ♂s and 2 ♀s (cl 3.25 – 5.34 mm); MNRJ 31168; Brazil, Rio de Janeiro, Macaé; (22°22'13.65"S, 41°39'9.42"W); colls. Davanso, T.M. et al.; 01 September 2013 • 1 ♂ and 1 ♀ (cl 4.20 and 5.53 mm, respectively); DZ/UFRGS 7089; Brazil, Rio de Janeiro, Macaé; (22°22'13.65"S, 41°39'9.42"W);colls. Davanso, T.M. et al.; 01 September 2013.

##### Additional material.

• > 30 ind. (not measured); CCDB 3251; same data as holotype • > 50 ind. (not measured); CCDB 7624; Brazil, Rio de Janeiro, Macaé; (22°22'13.65"S, 41°39'9.42"W); colls. Davanso, T.M. et al.; 01 September 2013.

##### Comparative material.

*Acetesa.americanus*: • 7 ind.; CCDB 6320; Brazil, Rio Grande do Norte, Baía Formosa; (06°21'11.6"S, 35°00'1.9"W); colls. Lopes, M., Carvalho-Batista, A.; 25 April 2014 • 2 ♀s (cl 3.6 and 5.1 mm); MZUSP 21210; Brazil, Alagoas, Maceió; 27/06/1989 • > 15 ind.; CCLC 258; Brazil, Espírito Santo, Anchieta, col. Braga, A.C.A.; 01 January 2014 • 10 ind.; CCDB 7626; Brazil, Rio de Janeiro, Macaé; (22°22'13.65"S, 41°39'9.42"W); colls. Davanso, T.M. et al.; 01 September 2013 • >10 ind.; CCLC 253; Brazil, São Paulo, Ubatuba, col. Costa, R.C.; 02 October 2014 • 10 ind.; CCDB 4939; Brazil, São Paulo, São Vicente, col. Castilho, A.L.; 03 September 2012 • 2 ♀s (cl 5.10 and 4.80 mm); CCLC 257; Brazil, Santa Catarina, Penha, coll. Davanso, T.M.; 24 June 2014.

*Acetesa.carolinae*: • 2 ♂s and 3 ♀s; ULLZ 3274; United States, Gulf of Mexico, Louisiana; coll. Forman, W.W.; 31 October 1972.

##### Diagnosis.

Rostrum acuminate, acute; median ridge with strong posterior tooth. Carapace smooth on surface, except for post-orbital and hepatic spine. Hepatic spine present in males, external part petasma not exceeding base of capitellum; inferior antennular flagellum with 10 articles. Concavity of anterior margin of genital sternite in females forming very deep arch.

##### Description.

**Figure 1.**
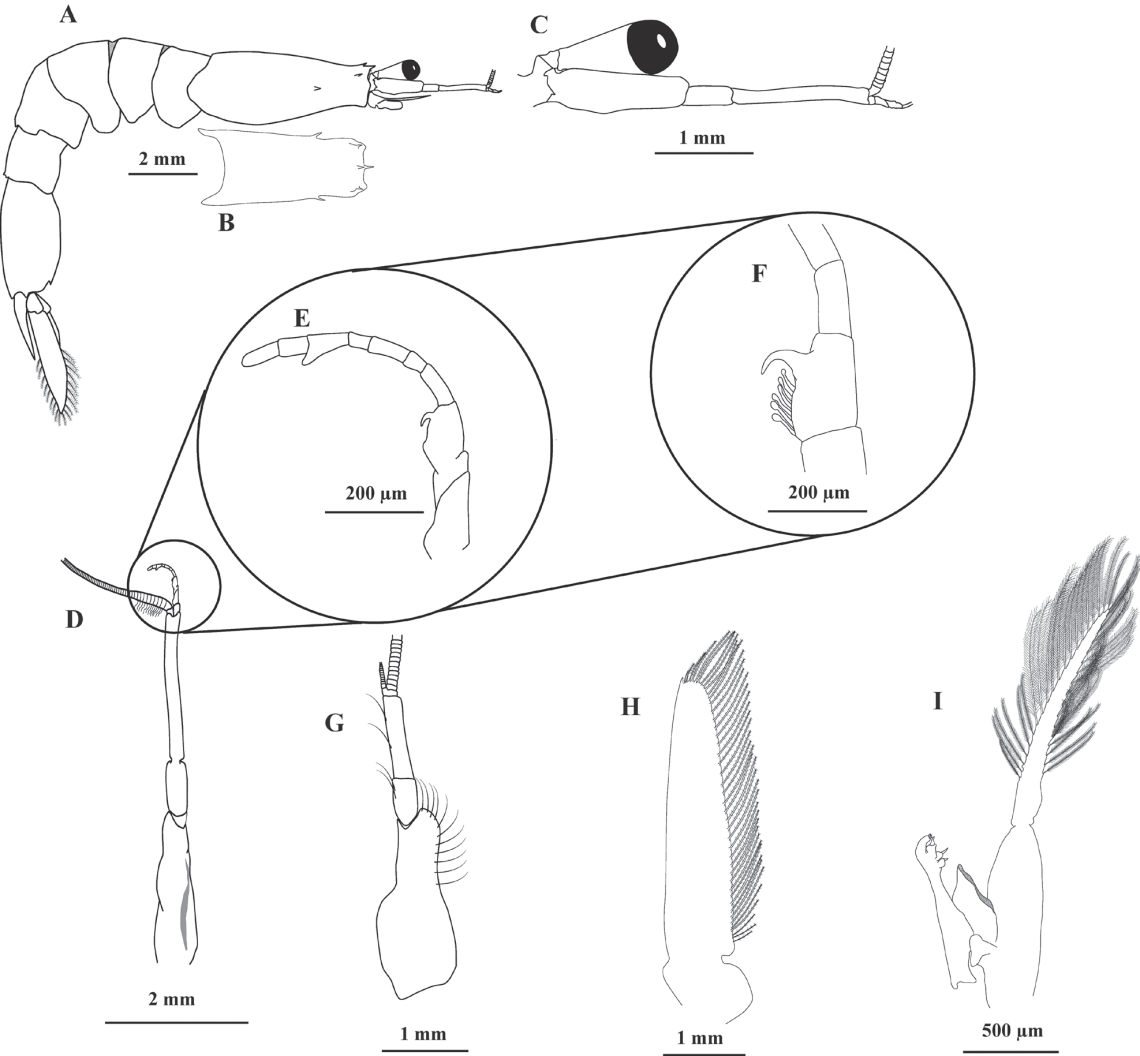
*Acetesmaratayama* sp. nov. **A–F, H–I** male paratype, Brazil, São Paulo, Cananéia (CCDB 7958) **G** female, paratype, Brazil, São Paulo, Cananéia (CCDB 7958) **A** lateral view **B** carapace, dorsal view **C** right antennular peduncle and ocular peduncle, lateral view **D** right antennular peduncle, dorsal view **E** lower antennular flagellum, lateral view **F** proximal part of lower antennular flagellum, lateral view **G** right antennular peduncle, dorsal view **H** scaphocerite, dorsal view **I** first pleopods and petasma, lateral view.

**Male.** The rostrum (Fig. 1A, B) is acuminate, acute; the median ridge has a strong posterior tooth. There is a small supraorbital spine on each side above the eyes, near the face. The hepatic spine is present (Fig. 1A). Quite large eyes do not exceed the posterior margin of the first antennular article (Fig. 1A, C). Antennule with long peduncle; very elongated third article, which is approximately three times longer than the inner margin of the second article, similar to the size of the first article (Fig. 1D); the first article in females is twice the length of the third article and approximately 4.5× longer than the second article; the inner distal lateral margin of the first article presents simple setae in the anterior half (Fig. 1G); males with inferior antennular flagellum have 10 articles; there is no clasping organ; males’ thickened proximal 3-article portion occupies less than half of the flagellum; third article has 6 obtuse spinules similar to fingers, and 1 procurved and robust projection (Fig. 1E, F); article 8 has a projection similar to a lobe in the inner lateral part of the article’s distal region (Fig. 1E). Antennal scale extending to the middle of the second antennular peduncle article with small spine on the anterior external portion (Fig. 1A, H).

**Figure 2.**
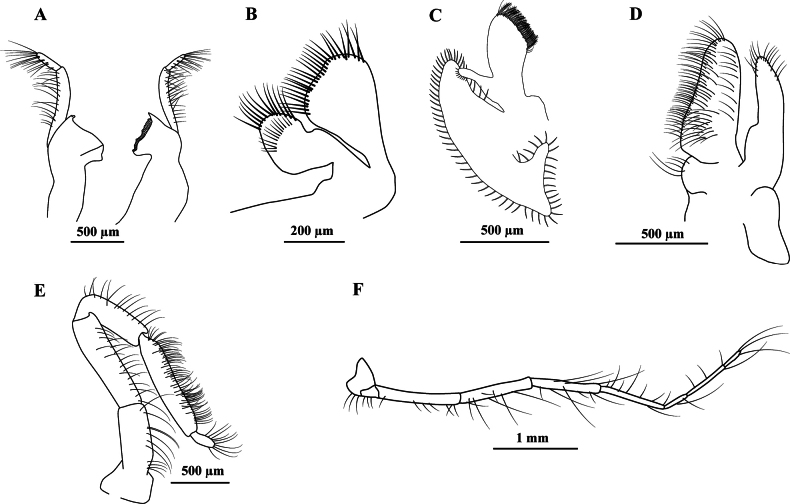
*Acetesmaratayama* sp. nov., male paratype, Brazil, São Paulo, Cananéia (CCDB 7958) **A** right and left mandible, lateral view **B** first maxilla, dorsal view **C** second maxilla, dorsal view **D** first maxilliped, dorsal view **E** second maxilliped, lateral view **F** third maxilliped, lateral view.

Mandible with biarticulated palp; first article of the palp 3× longer than the second article (Fig. 2A); first maxilla without palp (Fig. 2B); second maxilla with one single undivided lobe (Fig. 2C); first maxilliped without palp (Fig. 2D); second maxilliped with 5 articles (Fig. 2E); third maxilliped exceeding half of the antennal scale, without reaching the distal margin of the antennal scale (Fig. 2F).

**Figure 3.**
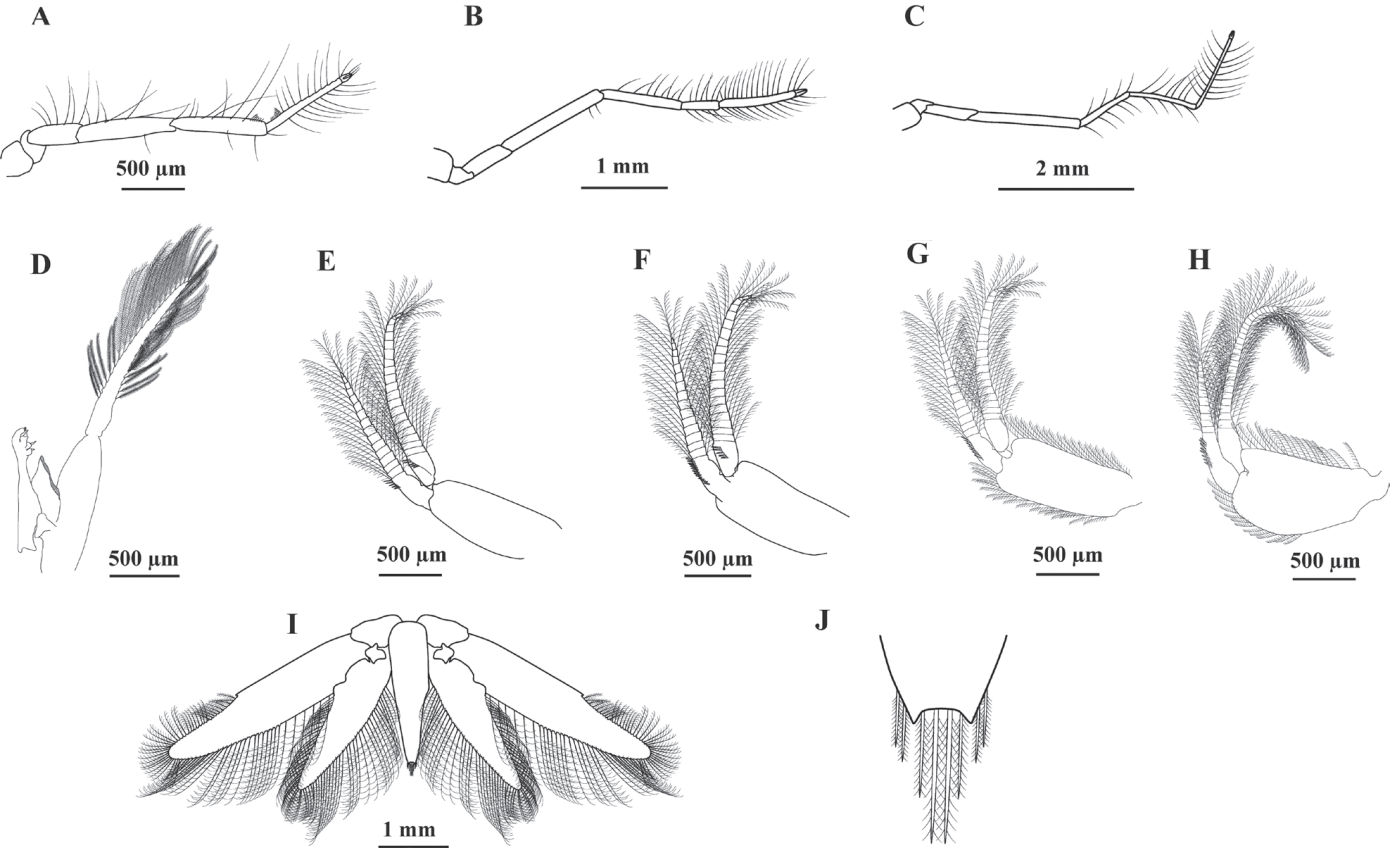
*Acetesmaratayama* sp. nov., male paratype, Brazil, São Paulo, Cananéia (CCDB 7958) **A** first pereiopod, lateral view **B** second pereiopod, lateral view **C** third pereiopod, lateral view **D** first pleopod with petasma, lateral view **E** second pleopod, lateral view **F** third pleopod, lateral view **G** fourth pleopod, lateral view **H** fifth pleopod, lateral view **I** uropod and telson, dorsal view **J** apex of telson, dorsal view.

The first 3 pairs of pereiopods are elongated and have a small chela (Fig. 3A–C). Fourth and fifth pereiopods were completely absent, except for a pair of protuberances (genital thighs) in males. The sixth segment of the pleon is longer than the others (Fig. 1A). Slender pleopods, the hind ones, are a little stockier. First pair with one single branch, with sexual appendages in males (Fig. 3D) - the remainder has two appendages (Fig. 3E–H). Pleopods with a row of spines on the basal articles of the endopods and exopods. PL2 with 5 spines on the outer margin of the endopod basal joint and 5 spines on the inner margin of the exopod basal joint (Fig. 3E); PL3 with 12 spines on the outer margin of the endopod and with 5 ones on the outer margin of the exopod (Fig. 3F); PL4 with 7 spines on the external margin of the endopod (Fig. 3G) and PL5 with 8 spines on the external margin of the endopod (Fig. 3H). Telson shorter than the anterior segment, long triangularly truncated at the tip (Fig. 3I). Uropods significantly longer than the telson, external branch much longer than the internal one, with a thin tooth on the external edge closer to the tip (Fig. 3I). The uropod exopod is 4.5 times longer than it is wide; a small spine on the outer margin in the 1/3^rd^ portion separates the ciliated portion from the non-ciliated portion (Fig. 3I). Telson apex is truncated; lateral margins are often curved inwards and form two short teeth between which the slightly convex posterior margin is found; there are 4 bristles between the terminal teeth, the two median ones are larger than the outer teeth, and two equal-sized bristles are external to the terminal teeth (Fig. 3J).

**Figure 4.**
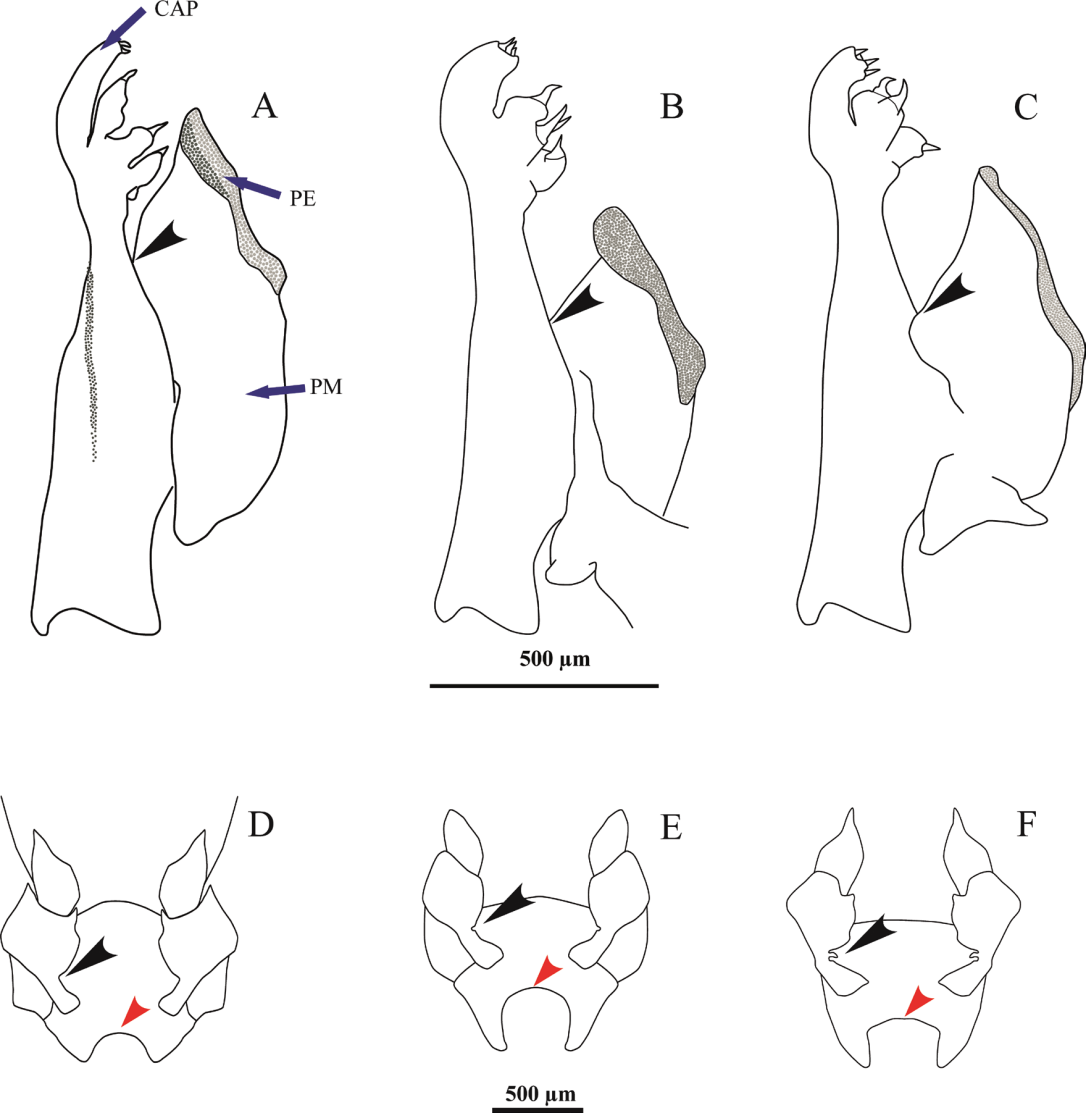
*Acetesamericanus***A, D**, male and female, Brazil, Rio de Janeiro, Macaé (CCDB 7626); *A.maratayama* sp. nov. **B, E**, male and female holotype, Brazil, São Paulo, Cananéia (CCDB 7957); *A.carolinae***C, F**, male and female, United States, Gulf of Mexico, Louisiana (ULLZ 3274) **A** petasma lateral view, and **D** genital sternite ventral view **B** petasma lateral view, and **E** genital sternite, ventral view **C** petasma lateral view, and **F** genital sternite, ventral view. Cap, capitulum; PE, pars externa; PM, pars media. In males, black arrows indicate the insertion of the pars externa into the pars media of the petasma. In females, black arrows indicate the inner margin of the thigh of the third pair of pereiopods, and red arrows indicate the curvature of the genital sternite.

**Males**. *Acetesmaratayama* sp. nov. is very similar to the other two described Atlantic species (*A.a.americanus* and *A.a.carolinae*), except for its different petasma and female genital sternite. Petasma pars externa in *A.maratayama* sp. nov. does not reach the base of the capitulum (Fig. 4B); the pars externa extends above the base of the capitulum in *A.a.carolinae* (Fig. 4C). On the other hand, it extends far beyond the capitulum base and reaches the middle portion of it in *A.americanus* (Fig. 4A). Pars externa insertion in *A.maratayama* sp. nov. is located in the middle section of the pars media (Fig. 4B, black arrow), similar to *A.a.carolinae* (Fig. 4C, black arrow). However, pars externa insertion in *A.americanus* is located close to the capitulum base (Fig. 4A, black arrow).

**Female.** The concave anterior margin of the genital sternite forms a very deep arch (Fig. 4E, red arrow) in comparison to *A.americanus* (Fig. 4D, red arrow), which has a shallow-arched concavity. The free sublateral projections by the margin’s sides are enclosed and taper to a defined point, besides being slightly curved. *Acetesa.carolinae* shape is similar to that of *A.maratayama* sp. nov.; however, the concavity of the genital sternite is not as deep, and the arch region is straight (Fig. 4F, red arrow). The thigh of the third pair of pereopods of *A.maratayama* sp. nov. accounts for most of the inner margin convex and presents a small indentation (Fig. 4E, black arrow). No tooth was found in its distal end. However, a large, oblong, acute process projects downwards the lower side of each thigh, close to the inner margin, and far forward and somewhat outward. *Acetesa.americanus* did not have an indentation (Fig. 4D, black arrow) and *A.a.carolinae* had a small projection (Fig. 4F, black arrow).

##### Habitat.

The species was collected by trawling in shallow waters in depths between 5 and 30 m. The bottom sediment type at the locality comprises medium and fine sand and has a salinity close to 26–28 ppt. It is considered the mesohaline area of the estuary (see Garcia et al. 2018 for environmental characterization of the Cananéia region’s bottom area).

##### Coloration in life.

Translucent like other species.

##### Type locality.

Brazil, São Paulo, Cananéia (24°59'55"S, 47°53'49"W).

##### Distribution.

Brazil, São Paulo, Cananéia (24°59'55"S, 47°53'49"W) and Rio de Janeiro, Macaé (22°22'13.65"S, 41°39'9.42"W).

##### Etymology.

The new species is named after the type locality, Cananéia, southern São Paulo state, Brazil. Maratayama is the old name of Cananéia recorded in the navigation log of the expedition from Portugal that arrived in the region in 1531. From the Tupi-Guarani language, Maratayama means a place where the land meets the sea or land of the sea (Mara = sea and Tayama = land).

##### Genetic sequences.

The previous genetic characterization and generated sequences obtained by Simões et al. [2023 – as “*Acetesamericanus* (Brazil 2)” - https://peerj.com/articles/14751/#supplemental-information] are updated and should be referred to as *Acetesmaratayama* sp. nov. The data, i.e., gene marker, geographic region, voucher catalogue collection and sequence accession number (GenBank), are: 16S Ribosomal RNA (16S) — Macaé/RJ: CCLC 0261 (OP035684 to OP035686), CCLC 0267 (OP035697); Cananéia/SP: CCLC 0262 (OP035687), CCDB 3251 (OP035688, OP035698 to OP035700); cytochrome *c* oxidase subunit I (COI) — Macaé/RJ: CCLC 0255 (OP060472), CCLC 0261 (OP060504 to OP060507), CCLC 0267 (OP060521 to OP060523); Cananéia/SP: CCLC 0262 (OP060508), CCDB 3251 (OP060509, OP060524 to OP060528). Some of these sequences were herein used to prepare the phylogenetic tree (Suppl. material 1).

##### Genetic distance.

16SrRNA gene: Intraspecific distances ranged from 0% (*A.americanus*, *A.maratayama* sp. nov. and *A.carolinae*) to 0.21% (*A.paraguayensis*) (Table 1). Interspecific distances between congeneric species ranged from 1.49 to 8.53% (Table 1). Regarding *A.maratayama* sp. nov., the smallest genetic distance observed was 0.85% with *A.carolinae*, 1.49% with *A.americanus* and the highest was 8.53% with *A.paraguayensis* (Table 1).

**Table 1.** Genetic distance values for the 16S rRNA gene between *Acetes* species distributed in the southwest Atlantic. The comparison is made between the same individuals used to build the phylogenetic trees, and the results show the minimum and maximum genetic differences recorded intra and interspecific.

	Species	1	2	3	4
1	*A.americanus*	0%			
2	*A.carolinae*	1.92%	0%		
3	*A.maratayama*	1.49%	0.85%	0%	
4	*A.paraguayensis*	8.32–8.53%	7.89–8.10%	8.32%–8.53%	0.21%

COI gene: Intraspecific distances ranged from 0 to 0.19% (*A.americanus* and *A.carolinae*), from 0 to 0.38 (*A.maratayama* sp. nov.), and from 0.57% (*A.paraguayensis*) (Table 2). Interspecific distances between congeneric species ranged from 4.78 to 19.89% (Table 2). Regarding *A.maratayama* sp. nov., the smallest genetic distance observed was with *A.americanus* (6.12–6.50%), followed by *A.carolinae* (7.65–8.63%), and the largest was with *A.paraguayensis* (19.50–19.89%) (Table 2).

**Table 2.** Genetic distance values for the COI gene between *Acetes* species distributed in the southwest Atlantic. The comparison is made between the same individuals used to build the phylogenetic trees, and the results show the minimum and maximum genetic differences recorded intra and interspecific.

	Species	1	2	3	4
1	*A.americanus*	0–0.19%			
2	*A.carolinae*	4.78–5.16%	0–0.19%		
3	*A.maratayama*	6.12–6.50%	7.65–8.63%	0–0.38%	
4	*A.paraguayensis*	19.31–19.89%	19.31–19.89%	19.50–19.89%	0.57%

##### Phylogenetic analyses.

**Figure 5.**
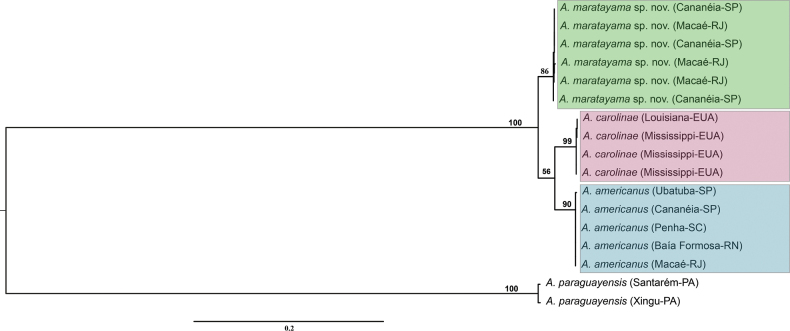
Pylogenetic reconstruction of *Acetes* based on concatenated markers 16S rRNA and COI. Phylogenetic tree of Bayesian inference for the *Acetes* species data with Bayesian posterior probabilities indicated (only posterior probabilities > 50% are shown).

The phylogenetic tree based on concatenated data (16S rRNA and COI) generated a similar topology found by Simões et al. (2023), with high support values. Two distinct clades were observed, one formed by *A.americanus* and *A.carolinae* and the sister clade formed by *A.maratayama* sp. nov. (Fig. 5)

##### Remarks.

*Acetesmaratayama* sp. nov. is closely related to *A.americanus* and *A.a.carolinae*, and it presents small morphological differences, mainly in reproductive structures. Furthermore, *A.maratayama* sp. nov. has 10 articles in the antennular flagellum, whereas *A.a.carolinae* has 9 articles, *A.binghami* Burkenroad, 1934a has 7 articles and *A.intermedius* Omori, 1975 has 13–14 articles. *Acetesmaratayama* sp. nov. is easily distinguishable from *A.binghami*, since the rostrum in this species does not have denticles behind the terminal tip, whereas the rostrum in *A.paraguayensis* has a strong tooth. There is a rudimentary denticle or hair minus one angular bend between this tooth and the end of the rostrum. The first article of the palp is 3 times longer than the second article. It is 5 times longer in *A.binghami.* The first article of the palp in *A.intermedius* is 2 times longer than the second article.

Historically, Burkenroad (1934b) recognized four *A.americanus* subspecies: *A.americanuscarolinae* (type locality: Beaufort Inlet, North Carolina, USA), *A.a.louisianensis* (type locality: Louisiana coast, from the Mississippi River West to Timbalier Island, Gulf of Mexico, USA), *A.a.limonensis* (type locality: Sweetwater River mouth, Limon Bay, Panama) and *A.a.americanus* (type locality: mouth of Tocantins River). However, *A.a.louisianensis* was synonymized with *A.a.carolinae* and *A.a.limonensis* was synonymized with *A.a.americanus* (DecaNet 2024). Holthuis (1948) states that subspecies *A.a.louisianensis* presents intermediate characteristics of other subspecies in this genus. They are not considered valid clinal variants. Burkenroad (1934b – Penaeidae from Louisiana, p. 132) states that:

“*Although I do not consider the differences here pointed out sufficiently certain or significant to require taxonomic recognition, if direct comparisons prove this to be desirable, I would suggest for Material from Louisiana with the subspecific name*Acetescarolinaelouisianensis.”.

This author also added important notes to Hansen’s (1919) description of *A.carolinae* (pp. 130–132).

Thus, several records show the geographic disjunction between the Gulf of Mexico and Panama and the well-documented vicariance processes in this region, which point out speciation between these regions and Western United States Atlantic (Coates and Obando 1996; Allmon 2001; Harrison 2004; Mantelatto et al. 2023). We are still not fully convinced that *A.a.louisianensis* is synonymous with *A.a.carolinae.* Therefore, more robust morphological analyses associated with molecular analyses must be carried out to help better understand these entities.

**Figure 6.**
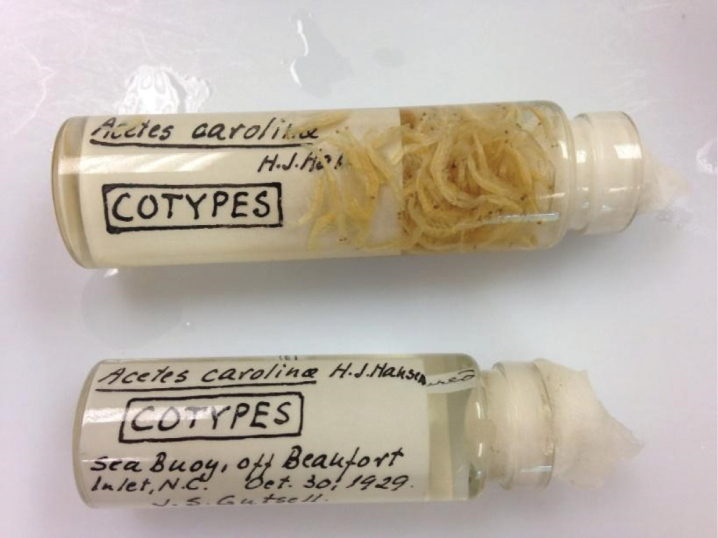
*Acetesa.carolinae* – type material of the original description by Hansen (1919) deposited at National Museum of Natural History, Smithsonian Institution, United States (USNM), Washington D.C., USA (USNM 74550). Photo credit: Kareen Reed and Sabrina Simões.

Individuals from the Western Atlantic (North Carolina - NC) were not included in the molecular analyses carried out by Simões et al. (2023), since they focused on species distributed within Brazil. It means that doubts about *A.a.carolinae* remain unresolved. Unfortunately, we did not have the opportunity to morphologically analyze the specimens (Fig. 6) identified as the cotype of *A.a.carolinae*, from USNM (74550). There is only one sequence (histone 3 gene) of *A.a.carolinae* from the North Carolina locality deposited in GenBank (KX216649) compared to our newly generated sequences of nuclear gene, histone 3 (H3), for individuals from Louisiana and Mississippi regions (ULLZ 14545 – Genbank PP816024, PP816025, PP816026). However, this gene’s DNA fragment (very conserved region) is not informative enough to identify congeneric species. Simões et al. (2023) recovered the lineage identified as “*A.americanus* (USA)”, which is formed by individuals from Louisiana and Mississippi, USA. Thus, doubts are raised about the likely validity/resurrection of subspecies *A.a.louisianensis*, which is strongly supported by the type locality being in the Gulf of Mexico. Further molecular analyses using other genes are necessary to elucidate the taxonomic status of *Acetes* species located in the Gulf of Mexico region and in North Carolina, named *A.a.carolinae*.

**Figure 7.**
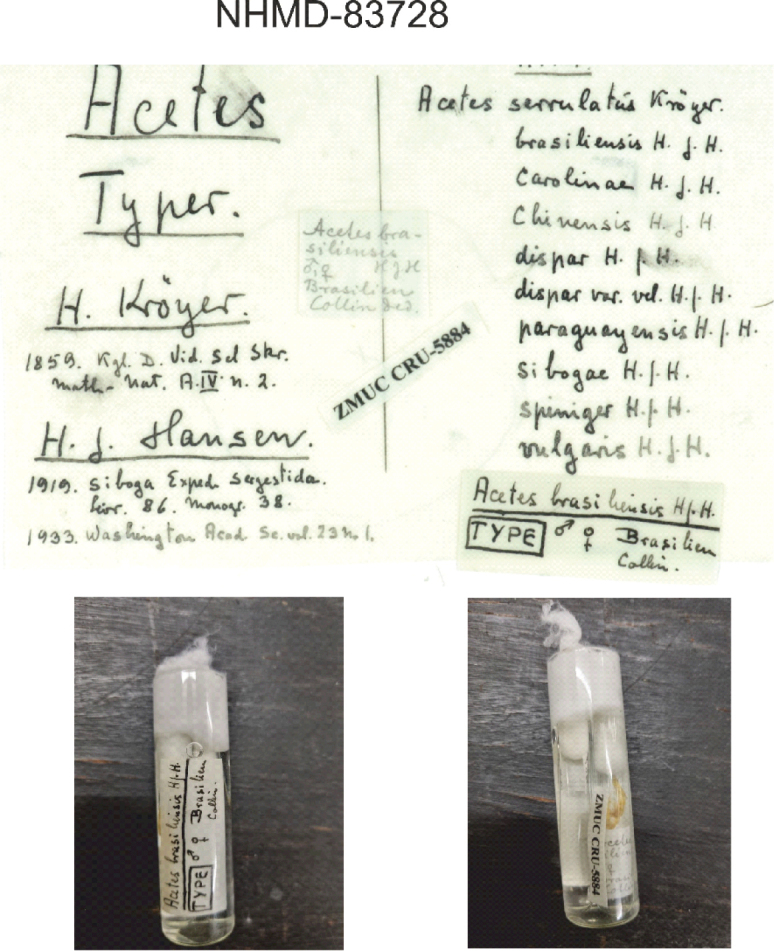
*Acetesbrasiliensis* - Type material of the original description by Hansen (1919) deposited at Natural History Museum of Denmark - University of Copenhagen (NHMD 83728). The number ZMUC-CRU-5884 on the label is the old museum catalogue number. Photos credit: Jørgen Olesen.

It is also important to recall that Hansen (1919) described *Acetesbrasiliensis* (p. 45–46, figs 1–7) collected from the Amazon River estuary. He mentioned the similarity to *A.americanus*, as described by Ortmann (1893), for collections from the mouth of Tocantins River, Brazil (Foz do rio Pará), which is very close to Amazon River. Despite a general description and undetailed figures, he emphasized that *A.brasiliensis* presented two features (length of third joint of the antennule and exopod of the uropod) making it impossible to refer *A.brasiliensis* to the species established by Ortmann. Burkenroad (1934b, p. 130), stated that:

“*The characters by which Hansen has distinguished*A.brasiliensis*from*A.americanus*seem of very uncertain importance. The differences in length of the ciliated part of the external margin of the exopod of the uropod, as those in other characters not mentioned by Hansen, are perhaps attributable to the obvious inaccuracy of Ortmann’s figure. That Ortmann failed to notice the elongation of the third segment of the antennular peduncle of the male of his species is no more astonishing than that Kishinouye failed to do so for* A. japonicus, *as Kemp has shown to be the fact.*”.

We had access (by photos) to the material (one male and one female – Fig. 7) used by Hansen to describe *A.brasiliensis*, due to the great help from Dr Jørgen Olesen (curator). They are deposited at the Natural History Museum of Denmark - University of Copenhagen (NHMD 83728). We agree with Burkenroad’s assertion and suggestion that *A.brasiliensis* is more likely synonymous with *A.americanus* after carefully analyzing the main characters.

## ﻿Conclusions

*Acetes* species from the Western Atlantic are morphologically similar to each other. When we integrated more robust morphological analyses, looking in greater detail at the petasma and genital sternite, into the previous multigene molecular analysis of Simões et al. (2023), we found significant differences and described a new species, *Acetesmaratayama* sp. nov. There is still taxonomic uncertainty regarding the specimens under the names *A.a.carolinae* and probably also regarding the synonymized *A.a.louisianensis*. At present, and pending future research, the name *Acetesmaratayama* sp. nov. should be adopted for Macaé City, Rio de Janeiro State, and for Cananéia City, São Paulo State, Brazil. *Acetesamericanus* should be adopted for Brazil (Northeastern Region: Rio Grande do Norte, Alagoas, Sergipe; Southeastern region: Espírito Santo, Rio de Janeiro, São Paulo; Southern region: Santa Catarina). *Acetesa.carolinae* is still unresolved and it most likely refers to specimens from the Western Atlantic, from North and South Carolina, given the doubts about specimens from the Gulf of Mexico and nearby areas.

### ﻿Key for American species of *Acetes*

**Table:** 

1	Rostrum without dorsal teeth	***Acetesbinghami* Burkenroad, 1934a**
–	Rostrum with one to two dorsal teeth	**2**
2	Rostrum with two dorsal teeth	***Acetesmarinus* Omori, 1975**
–	Rostrum with a single dorsal tooth	**3**
3	In males, the insertion of the pars externa is located near the base of the capitulum; the pars externa extends far beyond the base of the capitulum and reaches its middle portion. In females, the genital sternite has concavity’s anterior margin forming a very shallow arch	***Acetesamericanus* Ortmann, 1893**
–	In males, the insertion of the pars externa is located in the middle section of the pars media. In females, the genital sternite has concavity’s anterior margin forming a very deep arch	**4**
4	In males, petasma pars externa does not reach the base of the capitulum. In females, the genital sternite with the free sublateral projections by the margin’s sides are enclosed and taper to a defined point, besides being slightly curved	***Acetesmaratayama* sp. nov.**
–	In males, petasma pars externa extends above the base of the capitulum. In females, the concavity of the genital sternite is not so deep and the arch region is straight	***Acetescarolinae* Hansen, 1933**

## Supplementary Material

XML Treatment for Acetes maratayama

## ﻿Additional information

### Author ORCIDs

Gabriel L. Bochini https://orcid.org/0000-0001-9311-8419

Rogério C. Costa https://orcid.org/0000-0002-1342-7340

Fernando L. Mantelatto https://orcid.org/0000-0002-8497-187X

### Data availability

All of the data that support the findings of this study are available in the main text or Supplementary Information.

## Supplementary materials

Supplementary material 1 Selected species of *Acetes* and their respective localities, used in the construction of the phylogenetic hypothesis Data type docx Explanation note Sequences used in genetic analyses. 16S rRNA and COI marker numbers are available from NCBI Genbank. All data used are available in the supplemental information by Simões et al. (2023). CCDB = Crustacean Collection of the Department of Biology, Faculty of Philosophy, Science and Letters at Ribeirão Preto, University of São Paulo; CCLC = Crustacean Collection of the Laboratory of Biology of Marine and Freshwater Shrimp, São Paulo State University (UNESP), Bauru, Brazil; ULLZ = University of Louisiana Zoological Collection, Lafayette, USA. https://binary.pensoft.net/file/1128313This dataset is made available under the Open Database License (http://opendatacommons.org/licenses/odbl/1.0/). The Open Database License (ODbL) is a license agreement intended to allow users to freely share, modify, and use this Dataset while maintaining this same freedom for others, provided that the original source and author(s) are credited.Gabriel L. Bochini, Rogério C. Costa, Fernando L. Mantelatto
