# Supplementary material for: ﻿A new southern Atlantic cryptic marine shrimp species of Acetes (Decapoda, Sergestidae)
Source: Zookeys. 2024 Sep 4;1211:193–209. doi: 10.3897/zookeys.1211.128059 (PMC11391121; doi:10.3897/zookeys.1211.128059)
Supplement: Supplementary material 1 — Selected species of Acetes and their respective localities, used in the construction of the phylogenetic hypothesis [file zookeys-1211-193_article-128059__-s001.docx]

**Table S1.** Selected species of *Acetes* and their respective localities, used in the construction of the phylogenetic hypothesis. Sequences used in genetic analyses. 16S rRNA and COI marker numbers are available from NCBI Genbank. All data used are available in the supplemental information by Simões et al. (2023). CCDB = Crustacean Collection of the Department of Biology, Faculty of Philosophy, Science and Letters at Ribeirão Preto, University of São Paulo; CCLC = Crustacean Collection of the Laboratory of Biology of Marine and Freshwater Shrimp, São Paulo State University (UNESP), Bauru, Brazil; ULLZ = University of Louisiana Zoological Collection, Lafayette, USA

| Specimens | Collection | Locality | GenBank accession numbers | |
| --- | --- | --- | --- | --- |
|  |  |  | 16S | COI |
| *Acetes americanus* | CCLC 0253 | Ubatuba/SP/Brazil | OP035650 | OP060465 |
| *Acetes americanus* | CCLC 0254 | Macaé/RJ/Brazil | OP035655 | OP060470 |
| *Acetes americanus* | CCLC 0256 | Cananéia/SP/Brazil | OP035658 | OP060474 |
| *Acetes americanus* | CCDB 6320 | Baía Formosa/RN/Brazil | OP035661 | OP060477 |
| *Acetes americanus* | CCLC 0257 | Penha/SC/Brazil | OP035667 | OP060483 |
| *Acetes maratayama* sp. nov | CCLC 0261 | Macaé/RJ/Brazil | OP035684 | OP060504 |
| *Acetes maratayama* sp. nov | CCLC 0261 | Macaé/RJ/Brazil | OP035685 | OP060505 |
| *Acetes maratayama* sp. nov | CCLC 0261 | Macaé/RJ/Brazil | OP035686 | OP060506 |
| *Acetes maratayama* sp. nov | CCDB 3251 | Cananéia/SP/Brazil | OP035688 | OP060509 |
| *Acetes maratayama* sp. nov | CCDB 3251 | Cananéia/SP/Brazil | OP035699 | OP060525 |
| *Acetes maratayama* sp. nov | CCDB 3251 | Cananéia/SP/Brazil | OP035700 | OP060526 |
| *Acetes carolinae* | ULLZ 15593 | Lumcon/Louisiana/EUA | OP035679 | OP060498 |
| *Acetes carolinae* | ULLZ 14545 | Horn Island/Mississipi/EUA | OP035680 | OP060499 |
| *Acetes carolinae* | ULLZ 14545 | Horn Island/Mississipi/EUA | OP035681 | OP060500 |
| *Acetes carolinae* | ULLZ 14545 | Horn Island/Mississipi/EUA | OP035682 | OP060501 |
| *Acetes paraguayensis* | CCDB 4791 | Xingu/PA/Brazil | OP035672 | OP060490 |
| *Acetes paraguayensis* | CCDB 6200 | Santarém/PA/Brazil | OP035674 | OP060492 |
